# Co-administration of GF120918 significantly increases the systemic exposure to oral paclitaxel in cancer patients

**DOI:** 10.1054/bjoc.2000.1543

**Published:** 2001-01-01

**Authors:** M M Malingré, J H Beijnen, H Rosing, F J Koopman, R C Jewell, E M Paul, W W Ten Bokkel Huinink, J H M Schellens

**Affiliations:** Department of Medical Oncology, The Netherlands Cancer Institute/Antoni van Leeuwenhoek Hospital, Plesmanlaan 121, Amsterdam, CX, 1066, The Netherlands; Department of Pharmacy and Pharmacology, The Netherlands Cancer Institute/Slotervaart Hospital, Louwesweg 6, Amsterdam, EC, 1066, The Netherlands; Glaxo Wellcome, Research Triangle Park, Five Moore Drive, NC, 27709, USA; Division of Drug Toxicology, Faculty of Pharmacy, Utrecht University, Sorbonnelaan 16, Utrecht, CA, 3584, The Netherlands

**Keywords:** paclitaxel, oral administration, bioavailability, P-glycoprotein, GF120918

## Abstract

Oral bioavailability of paclitaxel is very low, which is due to efficient transport of the drug by the intestinal drug efflux pump P-glycoprotein (P-gp). We have recently demonstrated that the oral bioavailability of paclitaxel can be increased at least 7-fold by co-administration of the P-gp blocker cyclosporin A (CsA). Now we tested the potent alternative orally applicable non-immunosuppressive P-gp blocker GF120918. Six patients received one course of oral paclitaxel of 120 mg/m^2^ in combination with 1000 mg oral GF120918 (GG918, GW0918). Patients received intravenous (i.v.) paclitaxel 175 mg/m^2^ as a 3-hour infusion during subsequent courses. The mean area under the plasma concentration–time curve (AUC) of paclitaxel after oral drug administration in combination with GF120918 was 3.27 ± 1.67 μM.h. In our previously performed study of 120 mg/m^2^ oral paclitaxel in combination with CsA the mean AUC of paclitaxel was 2.55 ± 2.29 μM.h. After i.v. administration of paclitaxel the mean AUC was 15.92^ ^± 2.46 μM.h. The oral combination of paclitaxel with GF120918 was well tolerated. The increase in systemic exposure to paclitaxel in combination with GF120918 is of the same magnitude as in combination with CsA. GF120918 is a good and safe alternative for CsA and may enable chronic oral therapy with paclitaxel. © 2001 Cancer Research Campaign http://www.bjcancer.com


British Journal of Cancer (2001) **84**(1), 42–47
© 2001 Cancer Research Campaign
doi: 10.1054/ bjoc.2000.1543, available online at http://www.idealibrary.com on IDEAL®

http://www.bjcancer.com

### Co-administration of GF120918 significantly increases the systemic exposure to oral paclitaxel in cancer patients

**MM Malingré[1,2], JH Beijnen[1,2,4], H Rosing[2], FJ Koopman[2], RC Jewell[3], EM Paul[3], WW Ten Bokkel Huinink[1] and JHM Schellens[1,4]**

[1]Department of Medical Oncology, The Netherlands Cancer Institute/Antoni van Leeuwenhoek Hospital, Plesmanlaan 121, 1066 CX Amsterdam, The Netherlands; [2]Department of Pharmacy and Pharmacology, The Netherlands Cancer Institute/Slotervaart Hospital, Louwesweg 6, 1066 EC Amsterdam, The Netherlands; [3]Glaxo Wellcome, Five Moore Drive, Research Triangle Park, NC, 27709, USA; [4]Division of Drug Toxicology, Faculty of Pharmacy, Utrecht University, Sorbonnelaan 16, 3584 CA Utrecht, The Netherlands

**Summary** Oral bioavailability of paclitaxel is very low, which is due to efficient transport of the drug by the intestinal drug efflux pump P-glycoprotein (P-gp). We have recently demonstrated that the oral bioavailability of paclitaxel can be increased at least 7-fold by co-administration of the P-gp blocker cyclosporin A (CsA). Now we tested the potent alternative orally applicable non-immunosuppressive P-gp blocker GF120918. Six patients received one course of oral paclitaxel of 120 mg/m$^2$ in combination with 1000 mg oral GF120918 (GG918, GW0918). Patients received intravenous (i.v.) paclitaxel 175 mg/m$^2$ as a 3-hour infusion during subsequent courses. The mean area under the plasma concentration–time curve (AUC) of paclitaxel after oral drug administration in combination with GF120918 was 3.27 ± 1.67 μM.h. In our previously performed study of 120 mg/m$^2$ oral paclitaxel in combination with CsA the mean AUC of paclitaxel was 2.55 ± 2.29 μM.h. After i.v. administration of paclitaxel the mean AUC was 15.92 ± 2.46 μM.h. The oral combination of paclitaxel with GF120918 was well tolerated. The increase in systemic exposure to paclitaxel in combination with GF120918 is of the same magnitude as in combination with CsA. GF120918 is a good and safe alternative for CsA and may enable chronic oral therapy with paclitaxel. © 2001 Cancer Research Campaign http://www.bjcancer.com

**Keywords**: paclitaxel; oral administration; bioavailability; P-glycoprotein; GF120918

Paclitaxel is a potent anticancer drug with proven activity against a number of human solid tumours and has become standard treatment as single agent or in combination chemotherapy for the management of advanced breast, ovarian and non-small cell lung cancer (Rowinsky and Donehower, 1995; Natale, 1998). The intravenous (i.v.) administration of paclitaxel is, however, inconvenient for patients and associated with significant and unpredictable side-effects. Severe hypersensitivity reactions have been observed after i.v. infusion of paclitaxel and it is now well established that the pharmaceutical vehicle Cremophor EL contributes largely to this effect (Dye and Watkins, 1980; Weiss et al, 1990). Oral administration of paclitaxel is very attractive, because it is convenient and practical for patients and it may circumvent systemic exposure to the toxic vehicle Cremophor EL. Furthermore, oral administration may enable development of chronic treatment schedules resulting in sustained plasma concentrations above a pharmacological relevant threshold level. For paclitaxel a strong positive relationship has been reported between duration of the paclitaxel plasma concentration above 0.05 μM or 0.1 μM and myelosuppression (Huizing et al, 1993; Gianni et al, 1995; Ohtsu et al, 1995). In addition, Huizing et al (1997) have found, in a retrospective phase I/II study in patients with advanced non-small cell lung cancer, a significant survival benefit in patients who had an exposure duration of paclitaxel above 0.1 μM of more than 15 hours compared with patients who had a shorter duration of exposure above this cut off level. However, these data need confirmation in a prospective study. In view of increasing costs of anticancer therapy, oral treatment of paclitaxel is attractive, as oral administration eliminates the need for hospitalization, physician and nursing assistance and infusion equipment.

Up to now, oral administration of paclitaxel has not appeared feasible because of the low oral bioavailability (<10%) of the drug. Preclinical studies in mice have shown that the low oral bioavailability is due to efficient transport of the drug by the multidrug efflux pump P-glycoprotein (P-gp) abundantly present in the gastrointestinal tract (Schinkel, 1997; Van Asperen et al, 1998a). This became clear when we investigated the oral uptake of paclitaxel in mdrl a knock-out mice, which lack functional P-gp activity in the gut (Sparreboom et al, 1997). This mouse model revealed significant bioavailability of orally administered paclitaxel. In wild-type mice good bioavailability of orally administered paclitaxel was achieved when the drug was combined with cyclosporin A (CsA) or the cyclosporin analogue SDZ PSC 833, both efficacious blockers of P-gp (Van Asperen et al, 1997, 1998b). Based on our preclinical experiments we recently demonstrated the feasibility of oral administration of paclitaxel in cancer patients by concomitant administration of CsA. Co-administration of CsA resulted in a significant increase of at least 7-fold in the oral bioavailability of paclitaxel and plasma concentrations increased from negligible to therapeutic levels (Meerum Terwogt et al, 1998, 1999). In this study we tested the P-gp blocker GF120918 in combination with oral paclitaxel. GF120918, an acridone carboxamide derivative, is a potent inhibitor of P-gp. In in vitro models

*Received 22 May 2000*
*Revised 31 August 2000*
*Accepted 13 September 2000*

*Correspondence to:* MM Malingré

of P-gp inhibition, GF120918 is active at concentrations around 20 nM, which is about 100 fold more potent than CsA (Hyafil et al, 1993). Importantly, clinical studies of GF120918 with doses up to 1000 mg bid have shown no significant toxicities or side-effects of the drug (Ferry et al, 1998; Planting et al, 1998); it may therefore be a better candidate for clinical use, especially for repeated administration, than the immunosuppressive drug CsA. Based on the promising results of our preclinical studies with oral paclitaxel plus GF120918, revealing an approximately 7-fold increase in the systemic exposure in wild-type mice (Van Tellingen et al, 2000), we initiated this clinical study of orally administered paclitaxel in combination with oral GF120918.

### PATIENTS AND METHODS

### Patient population

Patients with a histologically confirmed cancer refractory to current therapies were eligible for the study. Previous radiotherapy or chemotherapy other than taxoid therapy was allowed, provided that the last treatment was at least four weeks prior to study entry and any resulting toxicities were resolved. Eligibility criteria included acceptable bone marrow function (white blood cells $> 3.0 \times 10^9$ $l^{-1}$; platelets $> 100 \times 10^9$ $l^{-1}$), liver function (serum bilirubin $\leq 25$ µmol $l^{-1}$; serum albumin $\geq 25$ g $l^{-1}$), kidney function (serum creatinine $\leq 160$ µmol $l^{-1}$ or clearance $\geq 50$ ml $min^{-1}$) and a World Health Organization (WHO) performance status $\leq 2$. Patients were not eligible if they suffered from uncontrolled infectious disease, neurologic disease, bowel obstruction or symptomatic brain metastases. Other exclusion criteria were concomitant use of known P-gp inhibitors and chronic use of H2-receptor antagonists or proton pump inhibitors. The study protocol was approved by the Medical Ethics Committee of the Institute and all patients gave written informed consent.

### Study design

Six patients received at one occasion paclitaxel orally 120 mg/m$^2$ in combination with a single oral dose of GF120918 of 1000 mg. At another occasion patients received intravenous (i.v.) paclitaxel administered as a 3-hour infusion at a dose of 175 mg/m$^2$. The oral and i.v. course were randomized. If it was considered to be in their best interest, patients continued on a 3-weekly schedule of i.v. paclitaxel. An oral paclitaxel dose of 120 mg/m$^2$ was selected for safety reasons because preclinical data of oral paclitaxel revealed that co-administration of a P-gp inhibitor and an oral paclitaxel dose can result in higher systemic exposure than after i.v. administration of the same dose (Van Asperen et al, 1997). An oral GF120918 dose of 1000 mg was selected because this dose was well tolerated and was expected to produce significant local P-gp blockade (Ferry et al, 1998; Planting et al, 1998). The i.v. formulation of paclitaxel (Taxol®, paclitaxel 6 mg ml$^{-1,}$ dissolved in Cremophor EL and ethanol 1:1 v/v, Bristol-Myers Squibb, Syracuse, NY, USA) was used for both i.v. and oral administration of paclitaxel. GF120918 (GG918, GW0918, Glaxo Wellcome, Research Triangle Park, NC, USA) (100 mg tablets) was ingested one hour prior to oral paclitaxel administration. As the absorption of GF120918 is improved after intake of a meal, the drug was ingested 30 minutes after a standard light breakfast. Patients fasted for two hours following oral paclitaxel intake. To prevent nausea and vomiting patients received oral granisetron 1 mg approximately 1 hour prior to oral paclitaxel administration. At all i.v. occasions, patients were premedicated with dexamethasone 20 mg orally 12 and 6 hours prior to, clemastine 2 mg i.v. 30 minutes prior to and cimetidine 300 mg i.v. shortly prior to paclitaxel administration.

### Patient evaluation

Pretreatment evaluation included a complete medical history and complete physical examination. Before each course, an interim history including concomitant medications taken, toxicities and performance status were registered and a physical examination was performed. Haematology was checked twice weekly after course 1 and 2 and weekly after subsequent courses. Blood chemistries including liver and renal function, serum electrolytes, total protein and albumin and glucose levels were checked weekly. All toxicities observed were graded according to the National Cancer Institute Common Toxicity Criteria (NCI CTC) (National Cancer Institute, 1988). Dose-limiting toxicities (DLT) were defined as grade 4 granulocytopenia of a duration of > 5 days, grade 4 thrombocytopenia of any duration or any grade 3/4 non-haematological toxicity except untreated nausea and vomiting. Tumour measurements were performed every other cycle, but initially after the first 2 i.v. courses. Responses were evaluated according to the WHO criteria (World Health Organization, 1979).

### Sample collection and analysis

Blood samples for pharmacokinetic analyses were collected during course 1 and course 2. For plasma paclitaxel and metabolite concentrations, blood samples of 5 ml each were collected at 0, 15, 30, 45, 60, 75, 90 minutes and 2, 3, 4, 7, 10, 24, 30 and 48 hours after oral intake of paclitaxel. During i.v. administration of paclitaxel a previously established limited sampling model was applied using 2 plasma concentration–time points at 1 and 8 hours after the end of the 3-hour infusion (Huizing et al, 1995a). Blood samples were centrifuged, plasma was separated and samples were immediately stored at –20˚C until analysis. Paclitaxel and metabolite concentrations were determined using a validated high performance liquid chromatography (HPLC) assay (Huizing et al, 1995b).

For GF120918 concentrations, blood samples of 7 ml each were collected on ice at 0, 30, 60, 90 minutes and 2, 3, 4, 5, 8, 11, 25, 31 and 49 hours after GF120918 intake. Blood samples were centrifuged at 4˚C, plasma was separated and samples were immediately stored at –20˚C until analysis. GF120918 concentrations were determined using a validated HPLC assay (Witherspoon et al, 1996).

### Pharmacokinetic analysis

Non-compartmental pharmacokinetic methods were applied to process the results (Gibaldi and Perrier, 1982). For orally administered paclitaxel, the maximal drug concentration ($C_{max}$) and time to maximal drug concentration ($T_{max}$) were obtained directly from the experimental data. The area under the plasma paclitaxel concentration–time curve was calculated by the trapezoidal rule up to the last measured concentration–time point (AUCt) and extrapolated to infinity using the terminal rate constant k (AUC). The time above the threshold concentrations of 0.05 µM and 0.1 µM

© 2001 Cancer Research Campaign

(T > 0.05 µM, T > 0.1 µM) was determined using linear interpolation. For i.v. administered paclitaxel the parameters AUC and T > 0.1 µM were determined using our previously established limited sampling model (Huizing et al, 1995a). Bioavailability of oral paclitaxel was calculated as the ratio of the AUC after oral and after i.v. administration with a correction for the difference in dose. Statistical analysis of the data was performed using the nonparametric Mann-Whitney U-test. The a priori level of significance was $P = 0.05$.

### RESULTS

### Patients and treatment

A total of six patients (3 males/3 females) was enrolled in the study. At study entry the median age of the patients was 58 years (range 49 to 65) with a median performance status of 1 (range 0–1). Primary tumour types included breast (1), non-small cell lung cancer (NSCLC) (2) and adenocarcinoma of unknown primary site (3). Three patients received oral paclitaxel in combination with GF120918 during the first course and i.v. paclitaxel during course 2. The other three patients received i.v. paclitaxel during course 1 and oral paclitaxel in combination with GF120918 during course 2. During all subsequent courses patients received i.v. administered paclitaxel.

The oral combination of paclitaxel and GF120918 was very well tolerated. No significant side-effects were seen after one course of oral paclitaxel in combination with GF120918. Haematological toxicities after oral administration of paclitaxel consisted of anaemia grade 1 (3 pts) and 2 (1 pt), which was often pre-existing, leukocytopenia grade 1 (1 pt) and 3 (1 pt) and granulocytopenia grade 2 (1 pt). Non-haematological toxicities after oral intake consisted of nausea grade 1 (1 pt) and 2 (1 pt), vomiting grade 2 (1 pt), arthralgia/myalgia grade 1 (2 pts), stomatitis grade 1 (1 pt), skin reactions grade 1 (1 pt), alopecia grade 1 (1 pt) and fatique grade 2 (1 pt). Toxicities clearly associated with GF120918 administration were not observed. During subsequent treatment with i.v. paclitaxel a toxicity pattern common to paclitaxel developed with anaemia, leukocytopenia, granulocytopenia, arthralgia/myalgia, nausea, vomiting, stomatitis, skin reactions, neurotoxicity and fatique as main toxicities. In this study no tumour responses were observed.

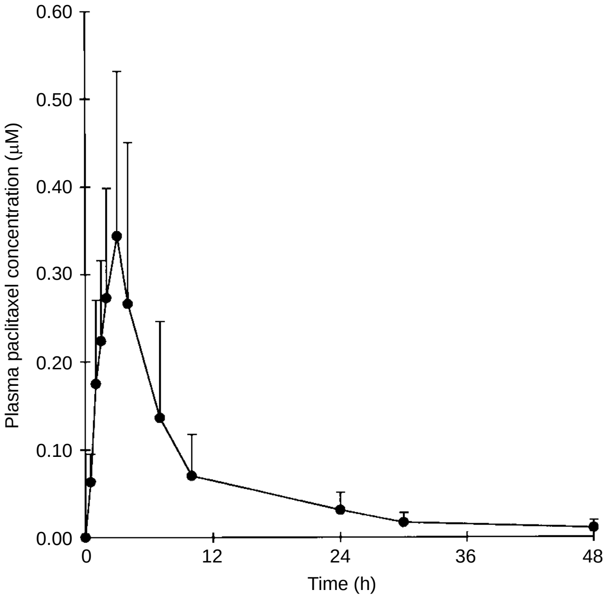

**Figure 1** Plasma concentration–time curve of oral paclitaxel at a dose of 120 mg/m² in combination with 1000 mg oral GF120918 ($n = 6$). Data are represented as means ± SD

### Pharmacokinetics

Table 1 summarizes the main pharmacokinetic parameters of oral and i.v. administered paclitaxel. The mean AUC value of paclitaxel in patients who received oral paclitaxel in combination with GF120918 was 3.27 ± 1.67 µM.h. There was no statistically significant difference in the paclitaxel AUC values between the patients who started with oral paclitaxel and GF120918 and those who received the drugs during the second course. The mean plasma concentration–time curve of oral paclitaxel in combination with GF120918 is shown in Figure 1. After i.v. administration, the mean AUC value of paclitaxel was 15.92 ± 2.46 µM.h which is in good agreement with earlier data (Huizing et al, 1993, 1997). The oral bioavailability of paclitaxel, calculated as the AUC after oral administration (120 mg/m²) divided by the AUC after i.v. administration (175 mg/m²) with a correction for the difference in dose, was 30 ± 15%. However, because of the pronounced non-linear

**Table 1** Pharmacokinetics of oral paclitaxel (120 mg/m²) in combination with GF120918 (1000 mg) and i.v. paclitaxel (175 mg/m² administered as a 3-hour infusion)

| | | Oral paclitaxel | | | | | | i.v. paclitaxel | |
|---|---|---|---|---|---|---|---|---|---|
| Patient | Course | AUC (µM.h) | $C_{max}$ (µM) | $T_{max}$ (h) | T > 0.1 µM (h) | T > 0.05 µM (h) | F (%) | AUC (µM.h) | T > 0.1 µM (h) |
| 1 | 2 | 4.56 | 0.48 | 3.0 | 8.8 | 15.1 | 36 | 18.59 | 22.3 |
| 2 | 1 | 4.36 | 0.33 | 3.0 | 15.3 | 25.0 | 49 | 13.01 | 12.1 |
| 3 | 1 | 5.23 | 0.62 | 3.0 | 9.6 | 23.9 | 41 | 18.59 | 20.9 |
| 4 | 2 | 1.11 | 0.17 | 1.9 | 2.6 | 3.9 | 11 | 14.90 | 13.8 |
| 5 | 2 | 1.92 | 0.20 | 3.1 | 4.2 | 6.1 | 17 | 16.87 | 16.6 |
| 6 | 1 | 2.41 | 0.33 | 3.0 | 4.3 | 6.7 | 26 | 13.54 | 13.9 |
| mean | | 3.27 | 0.36 | 2.8 | 7.5 | 13.5 | 30 | 15.92 | 16.6 |
| SD | | 1.67 | 0.17 | 0.5 | 4.7 | 9.3 | 15 | 2.46 | 4.2 |

AUC, area under the plasma concentration–time curve; $C_{max}$, maximal drug concentration; $T_{max}$, time to maximal drug concentration; T > 0.1 µM, time above 0.1 µM; T > 0.05 µM, time above 0.05 µM; F, bioavailability.

 © 2001 Cancer Research Campaign

**Table 2** Pharmacokinetics of oral paclitaxel (120 mg/m$^2$) in combination with 1000 mg GF 120918 and in combination with 15 mg kg$^{-1}$ cyclosporin A (the later data were taken from a previously published study (Malingré et al, 2000))

| | GF120918 (*n* = 6) | Cyclosporin A (CsA) (*n* = 3) |
|---|---|---|
| AUC (μM.h) | 3.27 ± 1.67 | 2.55 ± 2.29 |
| $C_{max}$ (μM) | 0.36 ± 0.17 | 0.31 ± 0.13 |
| $T_{max}$ (h) | 2.8 ± 0.5 | 3.7 ± 0.7 |
| T > 0.1 μM (h) | 7.5 ± 4.7 | 7.9 ± 8.0 |
| T > 0.05 μM (h) | 13.5 ± 9.3 | 13.0 ± 12.7 |

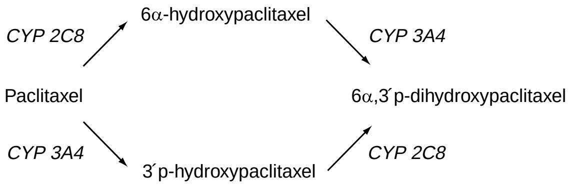

**Figure 2** Major metabolic pathways of paclitaxel pharmacokinetics of i.v. paclitaxel (Gianni et al, 1995; Sparreboom et al, 1996b), this calculation results in an underestimation of the true bioavailability. In a dose-finding study performed by Huizing et al (1997), a mean AUC of i.v. paclitaxel at a dose of 125 mg/m$^2$ of 6.8 μM.h was reported. Recalculation of the bioavailability of 120 mg/m$^2$ orally administered paclitaxel applying the dose-adjusted AUC found by Huizing et al (1997) provides a value of 50% for the oral bioavailability of paclitaxel in combination with GF120918.

The mean AUC and AUCt values of GF120918 were 13747 ± 9733 ng.h ml$^{-1}$ and 9428 ± 5431 ng.h ml$^{-1}$, respectively. AUCt values have been calculated because of the high per cent of the AUC extrapolated in two patients, i.e. 56% and 54% of the area under the curve. The mean maximum concentration of GF120918 was 434 ± 267 ng ml$^{-1}$ which was reached at 7.7 ± 2.5 hours after intake.

In Table 2 a comparison is made between the plasma pharmacokinetic parameters of paclitaxel after oral administration in combination with GF120918 and those of oral paclitaxel at the same dose but in combination with CsA. The latter data were taken from a study that has been performed previously at our Institute (Malingré et al, 2000). The mean paclitaxel AUC value in patients who received oral paclitaxel combined with GF120918 was 3.27 ± 1.67 μM.h and 2.55 ± 2.29 μM.h in patients who received oral paclitaxel in combination with CsA (not statistically significant). For the paclitaxel metabolites 6α-hydroxypaclitaxel, 3′p-hydroxypaclitaxel and 6α, 3′p-dihydroxypaclitaxel mean AUCt values after oral drug administration in combination with GF120918 (*n* = 5) were 0.40 ± 0.36, 0.36 ± 0.39 and 0.24 ± 0.34 μM.h respectively. Metabolite data of one patient could not be determined due to (unknown) interfering compounds in the analytical assay. After oral paclitaxel combined with CsA (*n* = 3) these values were 1.69 ± 2.71, 0.48 ± 0.50 and 0.88 ± 1.48 μM.h, respectively (these metabolite data have not been published before). The AUCt ratio for the metabolites 6α-hydroxypaclitaxel and 3′p-hydroxypaclitaxel was 1.1 (0.40/0.36) after oral drug administration with GF120918, whereas this ratio was 3.5 (1.69/0.48) when paclitaxel was combined with CsA. AUCt values have been calculated because extrapolation of the AUC could not be performed properly due to erratic profiles and the limited time that the metabolites could be detected.

### DISCUSSION

We have recently demonstrated that the poor oral bioavailability of paclitaxel can be increased at least 7-fold by co-administration of the P-gp blocker CsA (Meerum Terwogt et al, 1998, 1999). In this study we tested the potent alternative non-immunosuppressive P-gp blocker GF120918 in combination with oral paclitaxel.

The mean AUC value of paclitaxel achieved after oral administration in combination with GF120918 was 3.27 ± 1.67 μM.h, which is comparable to the AUC value achieved after oral paclitaxel administration in combination with CsA, i.e. 2.55 ± 2.29 μM.h (Malingré et al, 2000). Therefore, GF120918 is a good alternative for CsA in enhancing the oral bioavailability of paclitaxel. In both studies toxicities clearly related to the single dose administration of GF120918 or CsA were not observed. As the feasibility of oral paclitaxel administration will result in repeated dosing of either one of the P-gp blockers, the non-immunosuppressive agent GF120918 may be a better candidate for clinical use than the immunosuppressive drug CsA.

Preclinical studies of GF120918 with P-gp knock-out mice (Van Tellingen et al, 2000), which lack functional activity of P-gp, have shown similar pharmacokinetics of oral paclitaxel with or without co-administration of GF120918 and therefore indicate that the increase in systemic exposure to paclitaxel following GF120918 co-administration is solely due to blockade of P-gp. Our preclinical studies of CsA with wild-type mice (Van Asperen et al, 1998b), however, have shown higher AUC values of oral paclitaxel compared to those in P-gp knock-out mice without CsA (Sparreboom et al, 1997), indicating interference of CsA in uptake and elimination pathways of orally administered paclitaxel other than mediated by P-gp. For CsA, an important factor that may contribute to the increase in systemic exposure to oral paclitaxel is inhibition of paclitaxel metabolism; both paclitaxel and CsA are substrates for the cytochrome P450 (CYP) 3A4 isozymes (Shet et al, 1993; Harris et al, 1994) (Figure 2). In our previous study of oral paclitaxel in combination with CsA (Meerum Terwogt et al, 1999) we found that after oral drug administration (60 mg/m$^2$) in combination with CsA the relative contribution of formation of the metabolite 3′p-hydroxypaclitaxel was substantially lower than after i.v. administration, indicating inhibition of CYP 3A4 mediated paclitaxel metabolism by CsA. In the current study of oral paclitaxel in combination with GF120918 the AUCt ratio of 6α-hydroxypaclitaxel/3′p-hydroxypaclitaxel was 1.1, whereas this ratio was 3.5 when paclitaxel was combined with CsA, revealing a relative lower contribution of 3′p-hydroxypaclitaxel following CsA co-administration. These data also suggest inhibition of the CYP 3A4 mediated metabolic pathway of paclitaxel by CsA. Interpretation of the metabolite data should, however, be done with caution because of the small number of patients enrolled in each study and the very large interpatient variability in the metabolite data of paclitaxel. Furthermore, it is important to realise that inhibition of the CYP 3A4 mediated pathway will not necessarily result in prolonged exposure of active parent compound because drug not handled by CYP 3A4 might be handled by the CYP 2C8

*© 2001 Cancer Research Campaign*

pathway, which is, in general, the predominant metabolic pathway of paclitaxel.

In a control group of patients treated with oral paclitaxel (60 mg/m$^2$) but without co-administration of a P-gp blocker, a study which has been performed previously at our Institute, bioavailability of single agent oral paclitaxel was determined at 4% (Meerum Terwogt et al, 1998, 1999). In the current study, the oral bioavailability of paclitaxel (120 mg/m$^2$) in combination with GF120918 is determined at 30%. However, because i.v. paclitaxel shows pronounced non-linear pharmacokinetics (Gianni et al, 1995; Sparreboom et al, 1996b) these oral bioavailabilities, calculated using the AUC of i.v. paclitaxel at a dose of 175 mg/m$^2$, are underestimated. Using the pharmacokinetic data of i.v. paclitaxel at dose-levels of 100 mg/m$^2$ and 125 mg/m$^2$ (Huizing et al, 1997) at which less non-linearity is encountered, the apparent bioavailabilities are 6% for oral paclitaxel administered as a single agent (Meerum Terwogt et al, 1999) and 50% for orally administered paclitaxel in combination with GF120918.

An important pharmacokinetic parameter of paclitaxel is the time-period of exposure above a certain threshold concentration. Earlier data indicate a strong positive relationship between duration of the paclitaxel plasma concentration above 0.05 or 0.1 µM and pharmacological activity (Huizing et al, 1993, 1997; Gianni et al, 1995; Ohtsu et al, 1995). The feasibility of oral paclitaxel administration may enable the development of more chronic treatment schedules with sustained plasma concentrations above these pharmacological relevant threshold levels. However, it is important to discuss whether for orally administered paclitaxel these same threshold concentrations of 0.05 and 0.1 µM are relevant and should be pursued. Our previous studies of oral paclitaxel have shown that following oral administration of the drug the co-solvent Cremophor EL is not absorbed (Meerum Terwogt et al, 1998, 1999; Malingré et al, 2000). This is important, first of all, because systemic exposure to Cremophor EL can induce severe hypersensitivity reactions requiring extensive premedication (Dye and Watkins, 1980; Weiss et al, 1990). Consequently, paclitaxel can be administered orally without premedication, which has been done in the current study and without complications. On the other hand, however, several studies demonstrated that Cremophor EL is a potent modulator of multidrug resistance in vitro, and it has been hypothesized that this compound contributes to the clinical activity of paclitaxel (Woodcock et al, 1990; Webster et al, 1993). However, the extremely low volume of distribution of Cremophor EL (Sparreboom et al, 1998), the undetectable levels in (mouse) tissues (Sparreboom et al, 1996b), and the results in in vivo tumour-bearing models (Watanabe et al, 1996) suggest that this compound does not play a role in reversing P-gp mediated resistance to paclitaxel in vivo. Absence of systemic Cremophor EL after oral paclitaxel administration is also important because this compound is responsible for the non-linear pharmacokinetic behaviour of i.v. paclitaxel (Gianni et al, 1995; Sparreboom et al, 1996b). Cremophor EL increases the affinity of paclitaxel to plasma components which results in a more than proportional increase in plasma paclitaxel levels with increasing doses. However, studies in mice show that these higher 'total' drug levels in plasma do not result in higher drug levels in tissues (Sparreboom et al, 1996a). This pseudo-non-linearity of i.v. paclitaxel (Van Tellingen et al, 1999) has two important implications for the pharmacology of oral paclitaxel. First, as mentioned in the discussion above, it will result in a significant underestimation of the true bioavailability of oral paclitaxel. Second, the pseudo-non-linearity of i.v. paclitaxel implies that after oral administration, when Cremophor EL is not systemically present, plasma levels of paclitaxel represent a higher fraction of 'free' drug, which will result in enhancement of the availability of paclitaxel for the (tumour) tissues (Van Tellingen et al, 1999). Consequently, the optimal value of the threshold level may be lower for orally administered paclitaxel compared to i.v. paclitaxel; this needs further confirmation. Thus, comparison of paclitaxel plasma levels after oral and i.v. administration, without and with Cremophor EL in the systemic circulation, respectively, should be done with caution.

In summary, the P-gp inhibitor GF120918 is a good alternative for CsA administration in enhancing the oral bioavailability of paclitaxel. Importantly, GF120918 has no known immunosuppressive activity such as CsA and may therefore be a better candidate for clinical use, especially for repeated administration, than CsA.

### ACKNOWLEDGEMENTS

This work was supported by the Dutch Cancer Society (NKI project 98-1799).

### REFERENCES

Dye D and Watkins J (1980) Suspected anaphylactic reaction to Cremophor EL. *BMJ* **280**: 1353

Ferry D, Moore M, Bartlett NL, Fyfe D, Oza A, Fracasso PM, Kersey K, Wissel PS, Jewell RC and Paul EM (1998) Phase I and pharmacokinetic study targeting a 500 ng/mL plasma concentration of the potent multidrug resistance modulator GF120918 with doxorubicin in patients with advanced solid tumors. *Proc Am Soc Clin Oncol* **17**: 240a (abstract)

Gianni L, Kearns CM, Giani A, Capri G, Vigano L, Locatelli A, Bonadonna G and Egorin MJ (1995) Nonlinear pharmacokinetics and metabolism of paclitaxel and its pharmacokinetic/pharmacodynamic relationship in humans. *J Clin Oncol* **13**: 180–190

Gibaldi M and Perrier D (1982) Noncompartmental analysis based on statistical moment theory. In: Gibaldi M and Perrier D (eds) *Pharmacokinetics.* New York: Marcel Dekker, pp 409–417

Harris JW, Rahman A, Kim BR, Guengerich FP and Collins JM (1994) Metabolism of Taxol by human hepatic microsomes and liver slices: participation of cytochrome P450 3A4 and an unknown P450 enzyme. *Cancer Res* **54**: 4026–4035

Hyafil F, Vergely C, Du Vignaud P and Grand-Perret T (1993) *In vitro* and *in vivo* reversal of multidrug resistance by GF120918, an acridonecarboxamide derivative. *Cancer Res* **53**: 4595–4602

Huizing MT, Keung ACF, Rosing H, Van der Kuij V, Ten Bokkel Huinink WW, Mandjes I, Dubbelman AC, Pinedo HM and Beijnen JH (1993) Pharmacokinetics of paclitaxel and metabolites in a randomized comparative study in platinum-pretreated ovarian cancer patients. *J Clin Oncol* **11**: 2117–2135

Huizing MT, Van Warmerdam LJC, Rosing H, Ten Bokkel Huinink WW, Stewart MB, Pinedo HM and Beijnen JH (1995a) Limited sampling strategies for investigating paclitaxel pharmacokinetics in patients receiving 175 mg/m$^2$ as a 3-hour infusion. *Clin Drug Invest* **9**: 344–353

Huizing MT, Sparreboom A, Rosing H, Van Tellingen O, Pinedo HM and Beijnen JH (1995b) Quantification of paclitaxel metabolites in human plasma by high-performance liquid chromatography. *J Chromatogr B* **674**: 261–268

Huizing MT, Giaccone G, Van Warmerdam LJC, Rosing H, Bakker PJM, Vermorken JB, Postmus PE, Van Zandwijk N, Koolen MGJ, Ten Bokkel Huinink WW, Van der Vijgh WJF, Bierhorst FJ, Lai A, Dalesio O, Pinedo HM, Veenhof CHN and Beijnen JH (1997) Pharmacokinetics of paclitaxel and carboplatin in a dose escalating and sequencing study in patients with non-small cell lung cancer: A European Cancer Centre (ECC) trial. *J Clin Oncol* **15**: 317–329

Malingré MM, Meerum Terwogt JM, Beijnen JH, Rosing H, Koopman FJ, Van Tellingen O, Duchin K, Ten Bokkel Huinink WW, Swart M, Lieverst J and Schellens JHM (2000) Phase I and pharmacokinetic study of oral paclitaxel. *J Clin Oncol* **18**: 2468–2475

Meerum Terwogt JM, Beijnen JH, Ten Bokkel Huinink WW, Rosing H and Schellens JHM (1998) Co-administration of cyclosporin enables oral therapy with paclitaxel. *Lancet* **352**: 285

 © 2001 Cancer Research Campaign

Meerum Terwogt JM, Malingré MM, Beijnen JH, Ten Bokkel Huinink WW, Rosing H, Koopman FJ, Van Tellingen O, Swart M and Schellens JHM (1999) Co-administration of cyclosporin A enables oral therapy with paclitaxel. *Clin Cancer Res* **5**: 3379–3384

Natale RB (1998) Experience with new chemotherapeutic agents in non-small cell lung cancer. *Chest* **113**: (suppl 1) 32S–39S

National Cancer Institute, Division of Cancer Treatment and Bethesda, MD (1988) *Guidelines for Reporting of Adverse Drug Reactions*

Ohtsu T, Yasutsuna S, Tamura T, Miyata Y, Nakanomyo H, Nishiwaki Y and Saijo N (1995) Clinical pharmacokinetics and pharmacodynamics of paclitaxel: a 3-hour infusion versus a 24-hour infusion. *Clin Cancer Res* **1**: 599–606

Planting A, Van der Gaast A, Sparreboom A, Van der Burg MEL, De Boer M, Wissel PS, Jewell RC, Paul EM and Verweij J (1998) Phase I and pharmacokinetic study targeting a 100 ng/mL plasma concentration of the potent multidrug resistance modulator GF 120918 with doxorubicin in patients with advanced solid tumours. *Proc Am Soc Clin Oncol* **17**: 199a (abstract)

Rowinsky EK and Donehower RC (1995) Paclitaxel (Taxol). *N Engl J Med* **332**: 1004–1014

Schinkel AH (1997) The physiological function of drug-transporting P-glycoproteins. *Semin Cancer Biol* **8**: 161–170

Shet MS, Fisher CW, Holmans PL and Estabrook RW (1993) Human cytochrome P450 3A4: enzymatic properties of a purified recombinant fusion protein containing NADPH-P450 reductase. *Proc Natl Acad Sci USA* **90**: 11748–11752

Sparreboom A, Van Tellingen O, Nooijen WJ and Beijnen JH (1996a) Tissue distribution, metabolism and excretion of paclitaxel in mice. *Anti-Cancer Drugs* **7**: 78–86

Sparreboom A, Van Tellingen O, Nooijen WJ and Beijnen JH (1996b) Nonlinear pharmacokinetics of paclitaxel in mice results from the pharmaceutical vehicle Cremophor EL. *Cancer Res* **56**: 2112–2115

Sparreboom A, Van Asperen J, Mayer U, Schinkel AH, Smit JW, Meijer DKF, Borst P, Nooijen WJ, Beijnen JH and Van Tellingen O (1997) Limited oral bioavailability and active epithelial secretion of paclitaxel caused by P-glycoprotein in the intetestine. *Proc Natl Acad Sci USA* **94**: 2031–2035

Sparreboom A, Verweij J, Van der Burg ME, Loos WJ, Brouwer E, Vigano L, Locatelli A, De Vos AI, Nooter K, Stoter G and Gianni L (1998) Disposition of Cremophor EL in humans limits the potential for modulation of the multidrug resistance phenotype in vivo. *Clin Cancer Res* **4**: 1937–1942

Van Asperen J, Van Tellingen O, Sparreboom A, Schinkel AH, Borst P, Nooijen WJ and Beijnen JH (1997) Enhanced oral bioavailability of paclitaxel in mice co-treated with the P-glycoprotein blocker SDZ PSC 833. *Br J Cancer* **76**: 1181–1183

Van Asperen J, Van Tellingen O and Beijnen JH (1998a) The pharmacological role of P-glycoprotein in the intestinal epithelium. *Phramacol Res* **37**: 429–435

Van Asperen J, Van Tellingen O, Van der Valk MA, Rozenhart M and Beijnen JH (1996b) Enhanced oral absorption and decreased elimination of paclitaxel in mice cotreated with cyclosporin A. *Clin Cancer Res* **4**: 2293–2297

Van Tellingen O, Huizing MT, Nannan Panday VR, Schellens JHM, Nooijen WJ and Beijnen JH (1999) Cremophor EL causes (pseudo-)non-linear pharmacokinetics of paclitaxel in patients. *Br J Cancer* **81**: 330–335

Van Tellingen O, Bardelmeijer HA, Rosing H, Brouwer KR, Nooijen WJ and Beijnen JH (2000) Increased oral bioavailability of paclitaxel by GF120918 in mice through selective modulation of P-glycoprotein. *Proc Am Assoc Cancer Res* **41**: 673 (abstract)

Watanabe T, Nakayama Y, Naito M, Oh-hara T, Itoh Y and Tsuruo T (1996) Cremophor EL reversed multidrug resistance in vitro but not in tumor-bearing mouse models. *Anti Cancer Drugs* **7**: 825–833

Webster L, Linsenmeyer M, Millward M, Morton C, Bishop J and Woodcock D (1993) Measurement of Cremophor EL following Taxol: plasma levels sufficient to reverse drug exclusion mediated by the multidrug-resistance phenotype. *J Natl Cancer Inst* **85**: 1685–1690

Weiss RB, Donehower RC, Wiernik PH, Ohnuma T, Gralla RJ, Trump DL, Baker JR, Van Echo DA, Von Hoff DD and Layland-Jones B (1990) Hypersensitive reactions from Taxol. *J Clin Oncol* **8**: 1263–1268

Woodcock DM, Jefferson S, Linsenmeyer ME, Crowther PJ, Chojnowski GM, Williams B and Bertoncello I (1990) Reversal of the multidrug resistance phenotype with Cremophor EL, a common vehicle for water-insoluble vitamins and drugs. *Cancer Res* **50**: 4199–4203

Witherspoon SM, Emerson DL, Kerr BM, Lloyd TL, Dalton WS and Wissel PS (1996) Flow cytometric assay of modulation of P-glycoprotein function in whole blood by the multidrug resistance inhibitor GG918. *Clin Cancer Res* **2**: 7–12

World Health Organization (1979) *WHO Handbook for Reporting Results of Cancer Treatment*. Geneva, Switzerland

*© 2001 Cancer Research Campaign*
